# *Enterococcus* spp. Cell-Free Extract: An Abiotic Route for Synthesis of Selenium Nanoparticles (SeNPs), Their Characterisation and Inhibition of *Escherichia coli*

**DOI:** 10.3390/nano12040658

**Published:** 2022-02-16

**Authors:** Job T. Tendenedzai, Evans M. N. Chirwa, Hendrik G. Brink

**Affiliations:** Water Utilisation and Environmental Engineering Division, Department of Chemical Engineering, University of Pretoria, Pretoria 0002, South Africa; job.tendenedzai@tuks.co.za (J.T.T.); evans.chirwa@up.ac.za (E.M.N.C.)

**Keywords:** selenite, abiotic, cell-free extract, selenium nanoparticles, antibacterial

## Abstract

Selenite (SeO_3_^2−^), the most toxic and most reactive selenium (Se) oxyanion, can be reduced to elemental selenium (Se^0^) nanoparticles by a variety of bacteria, including *Enterococcus* spp. Previously, the orthodox view held that the reduction of SeO_3_^2−^ to Se^0^ by a wide range of bacteria was solely accomplished by biological processes; however, recent studies have shown that various bacterial strains secrete metal-reducing metabolites, thereby indirectly catalysing the reduction of these metal species. In the current study, selenium nanoparticles were synthesised from the abiotic reduction of selenite with the use of *Enterococcus* spp. cell-free extract. Once separated from the cell-free extract, the particles were analysed using Fourier-transform infrared (FTIR) spectroscopy, X-ray diffraction (XRD), Transmission electron microscopy (TEM) and a Zetasizer. The results revealed that the SeNPs were spherical in shape, containing both amorphous and crystalline properties, and the sizes with the highest frequency ranged close to 200 nm. Additionally, the obtained nanoparticles exhibited antimicrobial properties by directly inhibiting the viability of an *E. coli* bacterial strain. The results demonstrate not only the potential of abiotic production of SeNPs, but also the potential for these particles as microbial inhibitors in medical or similar fields.

## 1. Introduction

The emergence of nanotechnology in science and technology in recent years has dramatically transformed various industries. Materials with nanometre dimensions have properties and characteristics differing from bulk materials, which open new avenues for potential applications [1]. Agricultural, material science, environmental, biomedical and other fields have all slowly become dependent on what nanotechnology has to offer. Several metal nanoparticles such as silver (Ag), gold (Au) and selenium (Se) have shown promise in the biomedical field due to their enormous potential in the delivery of drugs, proteins and genes, as well as their anti-inflammatory and antioxidant effects [2]. A recent example of advancement in nanoparticle technology is the use of gold nanoparticles for the determination of uric acid in urine samples [3]. Elemental selenium, in the form of selenium nanoparticles (SeNPs), is also one of those materials which has also garnered interest in the biomedical field. Due to their antioxidant effects and ability to be incorporated in selenoproteins, SeNPs have been used in several therapeutic applications such as cancer and diabetes [1,2]. Moreover, from concentrations as low as 1 ppm, they have also been shown to inhibit both antibiotic-resistant gram-positive and gram-negative bacteria strains [2].

The study of selenium (Se) has gained momentum over the years because it has the unique attribute of being both essential and toxic to living organisms. In human beings, selenium concentrations of less than 55 μg·d^−1^ amount to selenium deficiency, whereas once it exceeds 400 μg·d^−1^ it can result in toxic effects [4]. Thus, the margin of safety between the essential and toxic levels of selenium is narrow, which is why minor variations in its environmental concentrations can have major impacts [2,5]. The guidelines for the maximum contaminant level for selenium in drinking water is set at 40 μg·Se/L and 50 μg·Se/L by the World Health Organization and the U.S. Environmental Protection Agency, respectively [6]. Although selenium is ubiquitous, anthropogenic activities such as phosphate mining, coal combustion and oil refining have led to selenium accumulating in both surface water and groundwater [7].

In the aquatic environment, selenium can exist in four oxidation states, namely the +6, +4, 0 and −2 states. It also occurs in organic forms [8,9]. The soluble oxyanions selenate (SeO_4_^2−^) and selenite (SeO_3_^2−^) are the most oxidised states of selenium. These have high solubility, which is why they are commonly found in surface waters [10]. SeO_4_^2−^ is the most prevalent, whereas SeO_3_^2−^ is the most toxic [8,11] and the most reactive, while usually being found in mildly oxidising acidic environments [9,12]. However, the oxyanions are both highly bioavailable and readily bioaccumulate in the food chain [10,13].

In contrast, elemental selenium (Se^0^) is naturally insoluble, displays a lower toxicological potential and can form selenium nanoparticles (SeNPs). SeNPs can be recovered and have potential uses, such as in the agricultural and pharmaceutical industries [14], for glass production and as detectors in mammographic instruments [15,16]

Several methods have been developed over the years for the remediation of selenium-laden waters. The remediation methods are in three categories, namely physical, chemical and biological techniques. Biological techniques are usually more favourable since they are deemed the most economically feasible option due to their low capital and operational and maintenance costs [17,18].

Microbial reduction can readily produce biogenic selenium nanoparticles (SeNPs) under both aerobic and anaerobic conditions. Moreover, the synthesis of SeNPs can occur at ambient temperature and pressure, making the process even more desirable. As already alluded to, microbial processes are relatively clean and eco-friendly methods. As a result, the biological synthesis of nanomaterials has drawn much attention [19]. There are a number of microbial species which are able to reduce selenite to elemental selenium as nanoparticles, but most of these utilise intracellular processes for SeNP production. Separating the intracellular nanoparticles from biomass for the purpose of selenium recovery can be achieved through processes such as cell lysis followed by filtration or centrifugation [20]. However, these processes are very energy-intensive and involve significant amounts of chemicals that can lead to further environmental contamination [21].

In contrast, there are species that can produce Bio-SeNPs extracellularly, thereby simplifying the recovery of the SeNPs. Despite this, in order to obtain the nanoparticles in their purest form, bacterial biomass often has to be separated from the SeNPs. This can be a drawback as it adds to incurred costs and lead time [22]. In this study, we propose the use of an abiotic system to circumvent this problem, as the nanoparticles can be synthesised in a cell-free extract, *viz.* a medium that initially contained active biomass which was subsequently removed, leaving active water-soluble biomolecules in solution. This process eliminates the requirement for separation of the selenium nanoparticles from the bacterial biomass altogether.

The first specific objective of this study is to demonstrate the reduction of selenite in a batch reactor using the cell-free extract of *Enterococcus* species. The second objective is to characterise the nanoparticles produced and explore possible applications by assessing how these inhibit a strain of the common food pathogen *E. coli*.

## 2. Materials and Methods

### 2.1. Culturing, Growing and Concentrating the Selenium-Reducing Bacteria

The bacterial culture used in the present work were *Enterococcus* spp. isolated from selenium-laden medium found in a laboratory at the University of Pretoria South Campus, South Africa [23]. For activation, the bacteria were aerobically cultivated in fresh Tryptone Soya Broth (TSB) (Oxoid Ltd., Basingstoke, UK) on a rotary shaker (Labotech, Midrand, South Africa) (28 °C, 24 h, 120 rpm). Thereafter, they were concentrated and harvested by centrifugation (6000 rpm, 25 °C, 5 min) before being utilised in the reduction experiments.

### 2.2. Chemicals, Culture Media and Solutions

All chemicals used were from Sigma-Aldrich (St. Louis, MO, USA) unless otherwise specified. Na_2_SeO_3_ was prepared as a 10 mM stock solution (stabilised with 300 mM NaOH). The mineral salt medium (MSM) was similar to the one used elsewhere [23], with slight modifications. The TSB for culturing the mixed culture was obtained from Oxoid Ltd. (Basingstoke, UK).

### 2.3. Abiotic Synthesis of SeNPs

In this study, the aerobic batch reduction of SeO_3_^2−^ was carried out in two stages: firstly, in the presence of bacterial biomass (biotic stage) for 1 h (in order to initiate the reaction); and secondly, in the absence of biomass by use of the cell-free extract (post biomass removal). The second stage was the abiotic system in which the nanoparticles were synthesised. Selenite was added as sodium selenite and varying initial selenite concentrations were reduced (1, 3 and 5 mM). The starting pH was between pH 8.5–9.5; the temperature was maintained at 35 ± 2 °C; the rotary speed was 120 rpm. The total experiment run time was 96 h. Samples were taken throughout the experiments and centrifuged before analysis. The selenite concentration was measured in the supernatant using the 940 Professional IC Vario ion chromatograph (Metrohm, Herisau, Switzerland) with separation column Metrosep C 6–250/4.0 (Metrohm, Herisau, Switzerland) and C 6- eluent- 8 mM oxalic acid (Metrohm, Herisau, Switzerland). Selenite which had been reduced to elemental selenium was concentrated in the pellet from centrifugation. For it to be quantified, it was first resuspended with 0.1% saline and washed before acid digestion (70% HNO_3_, 32% HCl, 60 min, 100 °C) in a thermo-reactor (Spectroquant^®^, Sigma-Aldrich, St. Louis, MO, USA). Total selenium in the digested sample was determined using a Perkin-Elmar AAS (Waltham, MA, USA) at 196.03 nm wavelength equipped with a 290 mA selenium lamp. All experiments were performed in triplicate unless otherwise stated. Some pellet samples were collected for further characterisation. These were purified by sequential centrifugation (10,000 rpm, 10 min) in 0.1% saline solution carbon-free, distilled, deionised water.

### 2.4. Cell-Free Extract Preparation

Viable bacterial biomass was present for the first hour of the reaction in order to initiate reduction, before being removed through centrifugation (6000 rpm, 5 min, 25 °C) and filtration using Whatman^®^ ME membranes for microbiological control (ME24/21 ST, diam. 50 mm, pore size 0.2 μm, Whatman PLC, Maidstone, UK). The remaining supernatant is what was referred to as the cell-free extract. Agar plates were streaked before and after the preparation of the cell-free extract. No growth was observed from the latter (after 24 h), further validating the absence of bacterial cells.

### 2.5. Characterisation of Selenium Nanoparticles (SeNPs)

The fabricated nanoparticles were characterised by a number of techniques. The Zetasizer Nano-ZS90 instrument (Malvern Instruments, Malvern, UK) was used for particle-size analysis. The particle-size distribution peak deconvolution was performed using the software package Origin 2021b (Originlab Corporation, Northampton, MA, USA). The FEGTEM: Jeol 2100 (Peabody, MA, USA) transmission electron microscope (TEM) was used for analysing the size and shape. The sphericities of the nanoparticles were estimated by image analysis of the TEM figures using the Software Package ImageJ (National Institutes of Health, Bethesda, MD, USA). The circularity index, *c* = 4*πS/P*^2^ (with *S* the measured surface and *P* the measured perimeter of the particle), was used as an approximation of the sphericity of the particles. A value of *c* = 1 indicates a perfect sphere or circle while *c* = 0 indicates a fully elongated shape. The crystallinity of the nanoparticles was studied by X-Ray Diffraction (XRD) with an X-ray diffractometer (Rigaku Corporation, Tokyo, Japan). The samples were prepared according to the standardised Panalytical backloading system, which provides nearly random distribution of the particles. Thereafter, the analysis was performed using a PANalytical X’Pert Pro powder diffractometer in θ–θ configuration with an X’Celerator detector and variable divergence and fixed receiving slits with Fe-filtered Co-Kα radiation (λ = 1.789 Å). The mineralogy was determined by selecting the best-fitting pattern from the ICSD database to the measured diffraction pattern, using X’Pert Highscore plus software. Fourier-transform infrared (FTIR) spectroscopy was used for the functional groups on the SeNPs and the FTIR spectrum was obtained in the wavenumber range of 4000–650 cm^−1^.

### 2.6. Antibacterial Properties

The antibacterial properties of the prepared nanoparticle samples were tested on the gram-negative *E. coli* bacterial strain obtained from a study which focused on chromium reduction [24]. Bacterial cultures were cultured on blood agar plates overnight at 37 °C. The colonies that had grown overnight were looped and inoculated into fresh broth from which a 10 mL sample would later be taken. In preparation for the inhibition tests, the SeNPs were recovered from the abiotic synthesis medium (see Section 2.3) by centrifugation (6000 rpm, 25 °C, 5 min) and filtration. Thereafter, the SeNPs were washed three times with saline before being allowed to air dry for 48 h.

The inhibition test was initiated by inoculating a 10 mL of sample in fresh broth and different concentrations of SeNPs were added. The MTT (3-(4,5-dimethylthiazol-2-yl)-2,5-diphenyltetrazolium bromide) tetrazolium assay, a popular tool in estimating the metabolic activity of living cells, was used to monitor activity over a 35 h period. The procedure for using the MTT assay as described by [25] was used.

## 3. Results and Discussion

### 3.1. SeO_3_^2−^ Reduction and SeNP Formation

**Figure 1:**
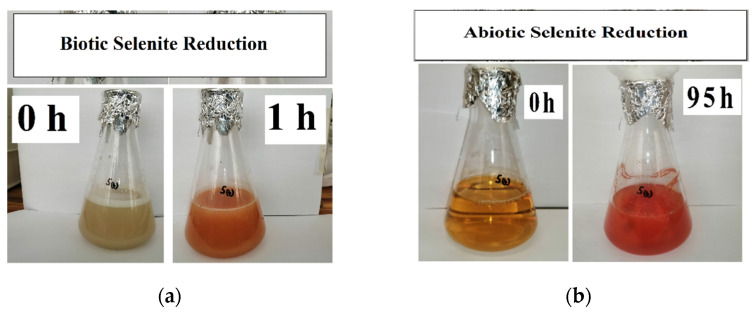
SeO_3_^2−^ reduction at the start and end of (**a**) biotic stage; (**b**) abiotic stage.

As aforementioned, reduction assays were conducted aerobically and divided into two stages, i.e., in the presence (for 1 h) and absence (for 95 h) of biomass. Figure 1 depicts the colour changes for these two stages. During the formation of red SeNPs, a garlic-like odour was generated, implying that the *Enterococcus* species were capable of volatilising selenium oxyanions as observed by Kagami et al. (2013) [26].

The generally observed trend was that selenite reduction was rapid for the first hour, prior to the removal of biomass. However, the percentage of selenite reduction depended on the initial selenite concentration at time 0 h. This is similar to what was observed by Dungan and Frankenberger (1998) and Tendenedzai et al. (2020) [27,28].

**Figure 2:**
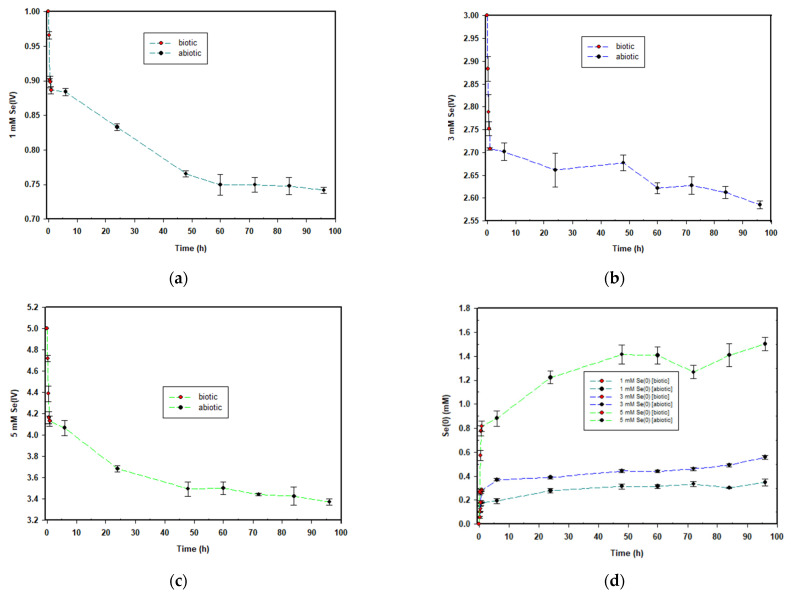
Time courses for the aerobic reduction of; (**a**) 1 mM, (**b**) 3 mM and (**c**) 5 mM SeO_3_^2−^ to Se^0^; and (**d**) the formation of Se^0^ across all the concentrations.

The selenite reduction profiles for the three concentrations are depicted in Figure 2. For the 1 mM concentration, 0.113 mM selenite was reduced during the biotic stage, translating to an average reduction rate of 0.113 mmol·(L·h)^−1^. A change in colour as depicted in Figure 1a corresponded with the observed reduction. For the abiotic stage, obtained results indicated that approximately 0.145 mM selenite was reduced, with an average reduction rate of 0.0015 mmol·(L·h)^−1^. As evident in Figure 1b, the intensity of the red colour in the MSM continued to increase, indicating the continued formation of elemental selenium and therefore that selenite reduction was still occurring in the cell-free extract. This was evident in all three concentrations.

For the 3 mM concentration, approximately 0.289 mM selenite was reduced during the biotic stage; the average reduction rate was 0.289 mmol·(L·h)^−1^. For the abiotic stage, obtained results indicated that approximately 0.192 mM selenite was reduced; the average reduction rate was 0.002 mmol·(L·h)^−1^.

Lastly, for the 5 mM concentration, approximately 0.868 mM selenite was reduced during the biotic stage; the average reduction rate was 0.868 mmol·(L·h)^−1^. For the duration of the abiotic stage, obtained results indicated that approximately 0.76 mM selenite was reduced; the reduction rate was 0.0008 mmol·(L·h)^−1^.

The common trend across the three selenite concentrations was that the higher the selenite concentration to be reduced, the greater the amount of selenite that was reduced, as well as the rate of selenite reduction. This is similar to what was observed in a previous study by Tendenedzai and Brink (2019) [29] in which a *Pseudomonas* strain was used. The explanation proposed by Tendenedzai and Brink (2019) for this observation is an increased biomass activity in response to increased selenite concentration [29]

The formation of elemental selenium nanoparticles is depicted in Figure 2d and mirrored the trend observed for selenite reduction. SeNP formation was rapid within the first hour when biomass was present, and it drastically reduced for the remainder of the 95 h during the abiotic stage.

The disproportion in the rates between the two stages described in this study shows that the presence of biomass influences both the rate of SeO_3_^2^**^−^** reduction and selenium nanoparticle formation. Moreover, it was observed that SeO_3_^2−^ reduction continued with the cell-free extract alone, after the biomass had been removed. This was taken as an indication of the presence of selenite-reducing biomolecules secreted into the supernatant by the bacterial cells prior to their removal. Saima Javed et al. (2015) showed that the cell-free extract of *Pseudomonas pseudoalcaligenes* was capable of reducing selenite. Moreover, these results were seen as confirmation that the strains released a reductase protein which reduced selenite extracellularly [30].

**Figure 3:**
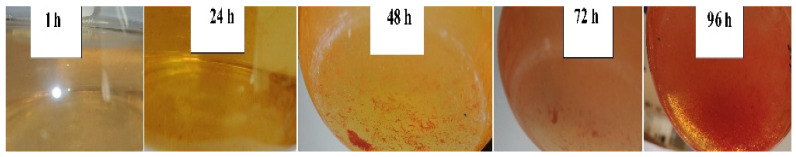
SeNP formation in the cell-free extract.

All three selenite concentrations showed a 48 h delay in the formation of visible SeNPs in the cell-free extract. Figure 3 shows the progression in SeNP formation. This is different when biomass is present as the formation of SeNPs is evident within the first 0.5 h [23,29]

Table 1 summarises the average selenium balance across the three concentrations. For the 1 and 3 mM SeO_3_^2−^ concentrations, the total was in excess by 9% and 3%, respectively, whereas for the 5 mM SeO_3_^2−^ concentration, a deficit of 2.5% was observed. The excess and deficit totals were considered to be negligible and attributed to either carry-over experimental errors or the possibility that volatisation might have taken place. Some bacterial strains are known to convert selenium oxyanions into DMDSe and DMSe after prolonged incubation [26].

### 3.2. XRD Analysis

**Figure 4:**
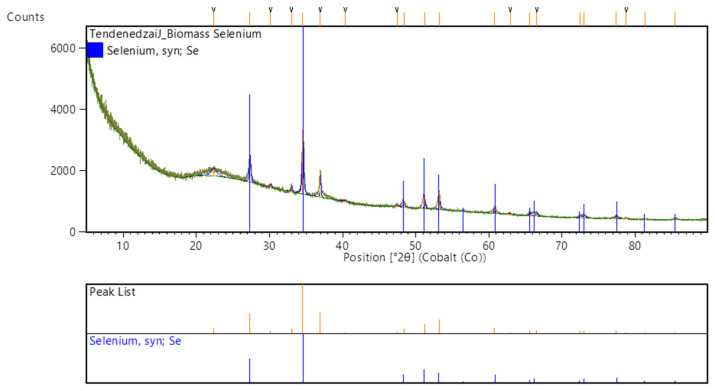
XRD pattern of selenium nanoparticles synthesised using the cell-free extract.

The analysed samples show a mixture of both crystalline and amorphous materials (Figure 4). This is because sharp peaks are typical of crystalline material, whereas amorphous material tends to have broadened ones.

To further validate the reached conclusion, it is imperative to understand the effect of temperature and pH on the crystallinity of produced biological SeNPs. In their study, Hageman et al. (2017) [31] concluded that at 40 °C combined with a pH > 7, crystalline bio-selenium particles are likely to be formed. The defined range in Hageman et al. (2017)’s study is largely similar to the experimental conditions during the first 1 h of the present study. It is only after the first hour that the pH decreases (<7). At this lower pH, more amorphous bio-selenium spheres are produced [31].

### 3.3. SeNP Morphology and Particle-Size Distribution

As alluded to earlier, a Zetasizer was used to analyse the particle-size distribution. The nanoparticles in each of the different concentrations were measured in order to investigate whether or not the initial concentration to be reduced had any bearings on the average particle size. The size of the Se^0^ particles grows until capping agents, such as proteins, polysaccharides, phospholipids or extracellular polymeric substances (EPS), inhibit further growth and aggregation [32,33]. Furthermore, proteins can restrict their size, with smaller particles observed at higher protein concentrations, while elongated incubation times can result in larger particle sizes (>200 nm) [34].

Upon analysis of the data from the Zetasizer, it became evident that different population distributions were present in each of the selenite concentrations reduced. When the frequencies were plotted, the graph was skewed to the left and thus not indicative of a normal distribution. In order to obtain a normal distribution, a log-normal distribution was applied on the particle sizes’ populations for the different concentrations.

**Figure 5:**
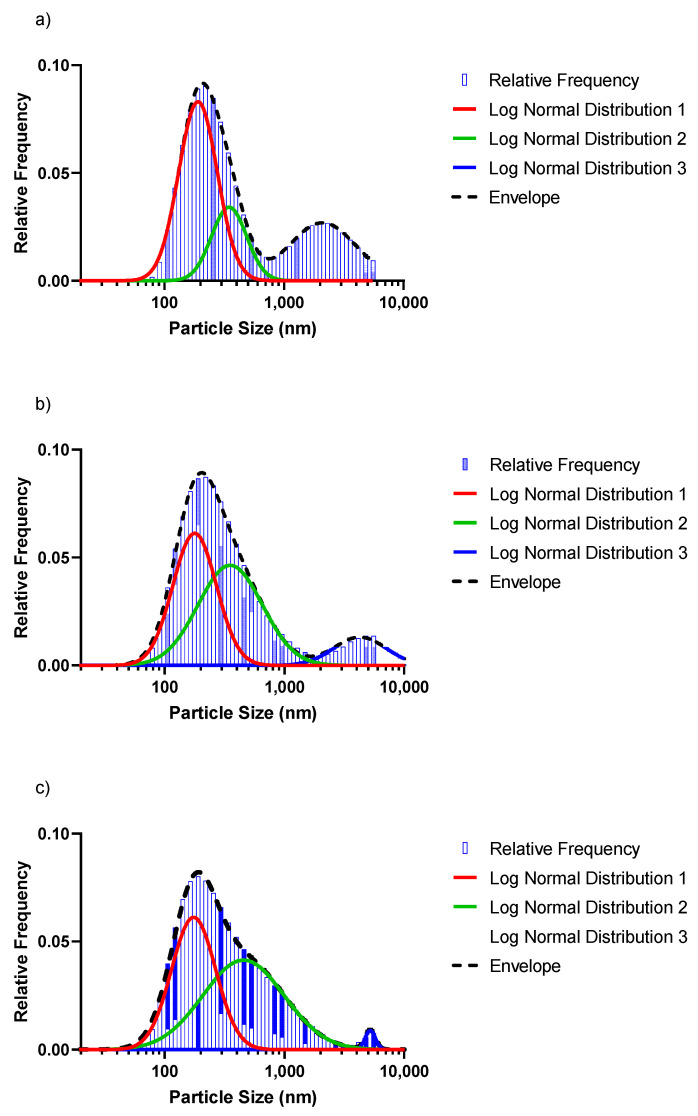
Log-normal distribution for the (**a**) 1 mM, (**b**) 3 mM and (**c**) 5 mM SeO_3_^2−^ concentrations, respectively.

Figure 5 shows the log-normal distribution for the 1 mM, 3 mM and 5 mM selenite concentrations. From Figure 5 can be seen that the red peaks, which are the particle sizes < 200 nm, had the highest frequency in the population regardless of the concentration. However, as the concentration to be reduced increased, the average particle sizes decreased as well. Table 2 summarises the fractions of particles in the three distinct populations, as well as the average particle sizes (*d*_50_) for the different distributions shown in Figure 5. The value for *d*_50_ defines the particle size at which the cumulative fraction of particles in a distribution reaches 50%.

The particles in the smaller size range (<200 nm) reduced in population (52.5% to 43.8%) and *d*_50_ (red peak) for increasing Se concentrations. The average particle sizes in the 200 nm range were 178.4, 166.6 and 163.2 nm for the 1 mM, 3 mM and 5 mM selenite concentrations, respectively. The green peak significantly increased in size, showing an increase in population fraction of 19.7%, 48.7% and 54.8% (1 mM, 3 mM and 5 mM), while the *d*_50_ remained nearly constant. The blue peaks shifted to the right, signalling an increase in the average particle sizes: 1940.1 nm (1 mM·Se), 3892.5 nm (3 mM·Se) and 4846.5 nm (5 mM·Se). In contrast, the size of the blue peak decreased significantly with a population fraction dropping from 29.5% (1 mM·Se) to only 1.5% for the 5 mM·Se. The results indicate that a significantly more homogenous size distribution (with a concomitant decreased overall *d*_50_) can be obtained with a higher Se concentration, with a concomitant decrease in average particle size.

**Figure 6:**
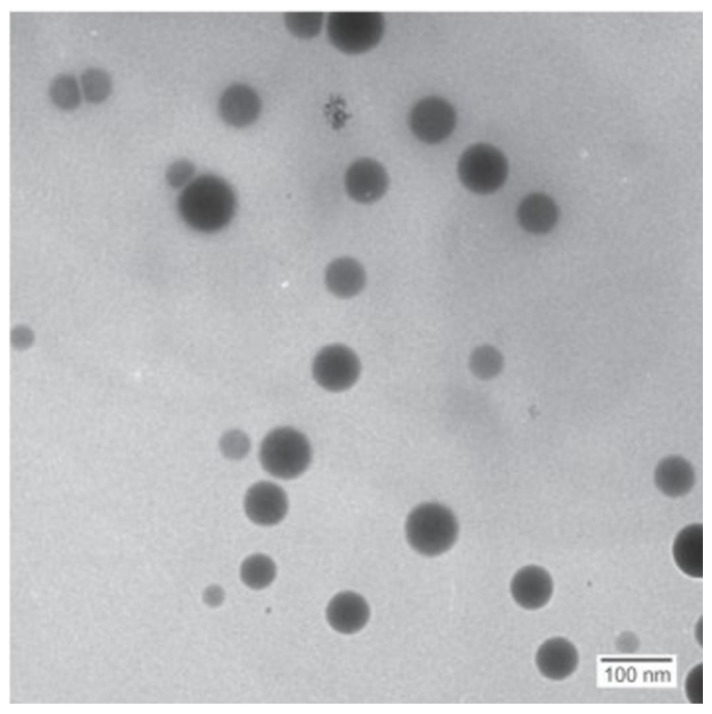
TEM images for SeNPs produced in the cell-free extract from the 5 mM concentration.

Figure 6 shows the TEM image from the 5 mM batch and indicates that the prepared nanoparticles were mostly spherical in shape. The results from the sphericity analysis performed using ImageJ showed circularity index values of (average ± standard deviation) *c* = 0.88 ± 0.04, 0.75 ± 0.04 and 0.89 ± 0.01 for the 1 mM, 3 mM and 5 mM runs, respectively. The results from the circularity measurements support the observed sphericity. The reduced sphericity of the intermediate selenium concentration run (3 mM) compared to that of the other runs (1 mM and 5 mM) is not clearly understood. However, it is known that the factors affecting nanoparticle sphericity are multifactorial and integrated and therefore would involve future study to elucidate the causes thereof.

### 3.4. FTIR Analysis

**Figure 7:**
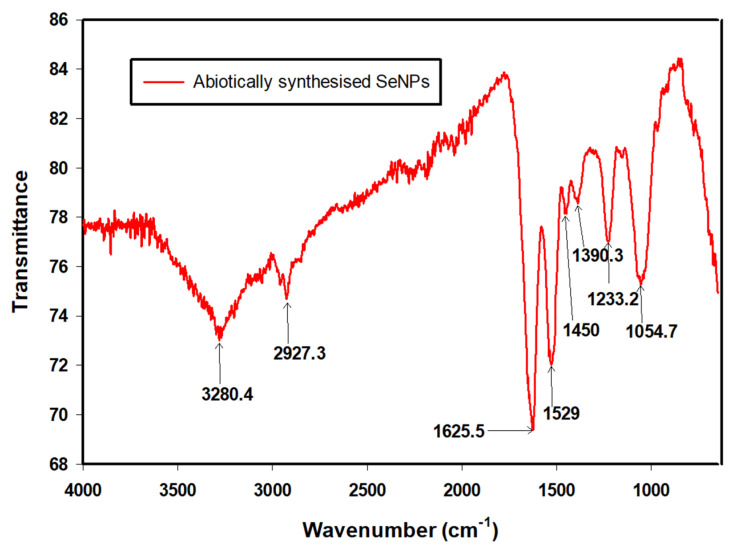
FTIR spectrum of selenium nanoparticles synthesised using the cell-free extract.

The synthesis of SeNPs and their functional groups were confirmed by FTIR spectroscopy. The spectrum (Figure 7) indicated the likely presence of macromolecules such as lipids, sugars, carbohydrates, nucleic acids and especially proteins that ensured the stability of the abiotic SeNPs [35]. The peaks were obtained between 4000 and 650 cm^−1^. The identities of the peaks are summarised in Table 3.

### 3.5. Antibacterial Efficiency of the SeNPs against E. coli

*E. coli* are gram-negative bacteria that are commonly present in the environment, foods and in the intestines of humans and animals. It is also one of the main pathogens responsible of nosocomial diseases [42]. An increase in antibiotic resistance of infections due to bacteria causing nosocomial diseases has been observed in recent times [43]. Therefore, it is imperative to develop new strategies to tackle this issue. Solutions can come from nanotechnology, with SeNPs having shown promise in a variety of biomedical applications [22].

In this study, preliminary investigations on the antibacterial efficiency of the abiotically produced selenium nanoparticles were conducted. The antibacterial test was performed on *E. coli* by using the MTT assay described earlier. Various concentrations of selenium nanoparticles were added to the broth already inoculated with the bacteria and incubated for 36 h. Metabolic activity assays were conducted periodically throughout the experimental period.

**Figure 8:**
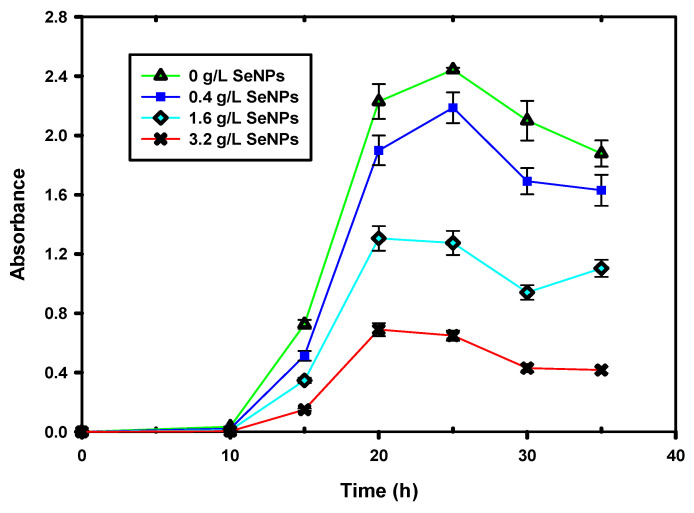
Cell viability evaluation in the presence of SeNPs.

Figure 8 shows the variation in metabolic activity inhibition due to SeNPs. When no nanoparticles had been added, the metabolic activity of *E. coli* was highest, and its decline was due to natural cell death. However, as the concentration of the nanoparticles increased, so did the reduced cell viability. It can be seen from Figure 8 that the highest concentration of nanoparticles used (3.2 g/L) showed the greatest inhibition.

Although total inhibition could not be achieved within the scope of this study, the results are still encouraging and point to a potential use for the nanoparticles. The mechanism of the cytotoxicity of SeNPs against bacteria are still unclear. However, it is known that many bacteria have a negatively charged surface and the zeta potential of the nanoparticles plays an important role due to this [44]. In this study, the gram-negative bacteria *E. coli* was used. Therefore, a likely reason for the limited inhibition of the bacteria could have been because of the strong repulsive forces existing between SeNPs and highly negatively charged bacteria such as *E. coli*. This is possibly due to the presence of a layer of negatively charged lipopolysaccharides in gram-negative bacterial cell walls, resulting in these being more negatively charged than gram-positive bacteria [45]. Therefore, in theory, the effectiveness for inhibition for the SeNPs on bacteria with a lower (or neutral) surface net charge, such as *S. aureus*, should be greater. This has been shown to be true in other studies [42].

In addition, the particle sizes of the nanoparticles may have had a bearing on the limited effectiveness of the inhibition. Typically, the suitable size range adapted to interact with bacteria would be <100 nm [46], which results in a large surface-to-volume ratio, thereby leading to greater efficiency.

## 4. Conclusions

This study presented an eco-friendly method for producing selenium nanoparticles using the cell-free extract of *Enterococcus* spp. The SeNPs, which exhibited high antibacterial activity by affecting the viability of *E. coli* cells, were relatively easy to recover due to the absence of biomass. This is a significant advantage as it eliminates costly processes of further purifying the SeNPs before use. FTIR results signalled the presence of proteins and polysaccharides among other bio-macromolecules capping the SeNPs, providing stability to these particles. The results from the study further showed that controlling the concentration of selenite in solution is important as it affects the particle-size distribution of the synthesised nanoparticles—an important consideration for scaling and implementation of the technology. The current study demonstrates the suitability of the abiotic system as a potential synthesis process for the economic production for SeNPs with antimicrobial application.

## Figures and Tables

**Table 1:** Average selenium balance across the 1, 3 and 5 mM SeO_3_^2−^ concentrations.

Concentration (mM)	Average SeO_3_^2−^ Remaining (mM)	Average Se^0^ Formation (mM)	Total Average Se (mM)
1	0.742	0.348	1.09
3	2.535	0.558	3.093
5	3.372	1.501	4.873

**Table 2:** Distribution properties of the respective particle-size distribution of abiotically synthesised SeNPs (Figure 5).

Selenium Concentration	Distribution 1 (Red Peak)		Distribution 2 (Green Peak)		Distribution 3 (Blue Peak)		Total Distribution (Dashed Line/Bars)	
	% of Total	*d*_50_ Predicted^1^	% of Total	*d*_50_ Predicted^1^	% of Total	*d*_50_ Predicted^1^	*d*_50_ Predicted ^1^	*d*_50_ Measured ^2^
1 mM	52.5	178.4	19.7	323.6	29.5	1940.1	267.2	278.8
3 mM	43.3	166.6	48.7	329.4	10.9	3892.5	247.3	244.9
5 mM	43.8	163.2	54.8	318.1	1.5	4846.5	248.5	246.5

^1^ As predicted from the distribution curves; ^2^ As measured directly by the Zetasizer.

**Table 3:** Wavenumbers of the main bands in the FTIR spectrum of abiotically synthesised SeNPs.

Wavenumber (cm^−1^)	Functional Groups	References
3280.4	N–H_2_, aminoacidic group	[36]
2927.3	C–H, C–H_2_ stretch, Alkanes, aliphatic groups, fatty acid aliphatic chains	[37]
1625.5	N–H stretch, Secondary amine, amide I	[38]
1529	C–N stretch, amide II band, alkanes	[36]
1450	–CH_2_/–CH_3_ (in proteins, lipids, polyesters, etc.)	[36,39]
1390.3	Carboxyl (–COO^−^) stretching vibration	[37,40]
1233.2	C–N stretch, amide III band, O–P–O	[36]
1054.7	Cyclohexane ring vibrations/Aromatic C–H in-plane bend/Aliphatic fluoro compounds, C–F stretch/Primary amine C–N stretch C–O, C–C, (In polysaccharides, proteins and polyesters)	[36,41]

## Data Availability

The data presented in this study are openly available in the University of Pretoria Research Data Repository at doi:10.25403/UPresearchdata.19169507 (accessed on 1 February 2022).
